# The Joint Crisis Plan: A Powerful Tool to Promote Mental Health

**DOI:** 10.3389/fpsyt.2021.621436

**Published:** 2021-03-19

**Authors:** Pierre Lequin, Pascale Ferrari, Caroline Suter, Marion Milovan, Christine Besse, Benedetta Silva, Philippe Golay, Charles Bonsack, Jérôme Favrod

**Affiliations:** ^1^Department of Psychiatry, University Hospital Centre, Lausanne, Switzerland; ^2^La Source, School of Nursing, HES-SO University of Applied Sciences and Arts of Western Switzerland, Lausanne, Switzerland

**Keywords:** mental health, joint crisis plan, shared decision making, recovery, therapeutic collaboration, self-management

## Abstract

**Purpose:** The Joint Crisis Plan (JCP) has received growing interest in clinical and research settings. JCP is a type of psychiatric advance statement that describes how to recognize early signs of crisis and how to manage crises. The purpose of the present study, to our knowledge the first to be conducted on this topic in the French-speaking context and to include inpatients, was to describe the content of JCPs and how they are perceived by patients and the providers.

**Methods:** The study used an exploratory, mixed, sequential method. Existing JCPs were retrospectively collected in several clinical contexts (hospital, community settings, and sheltered accommodation). Based on their analyses, we conducted semi-structured interviews including some rating scales on the perception of the JCPs among patients and providers in these settings. For the qualitative analyses, content analyses were conducted with a hybrid approach using NVivo 12 software. Data were double-coded and discussed with a third researcher until agreement was reached.

**Results:** One hundred eighty-four JCPs were collected retrospectively and 24 semi-structured interviews were conducted with 12 patients and 12 providers. No relatives could be included in the research process. The content of the studied JCPs was relevant and indicated that patients had good knowledge of themselves and their illness. Improvements in the quality of the therapeutic relationship, respect for patients' choices and wishes, and a greater sense of control of their illness were reported. The JCP was perceived as a very useful tool by patients and providers. Concerning JCP limitations, lack of staff training, difficulties with the shared decision-making process, and the poor availability of the JCPs when needed were reported.

**Conclusion:** The study highlights that JCPs may be used with patients suffering from a large variety of psychiatric disorders in different care settings. The JCP is perceived as very useful by both patients and providers. The promising results of this study support the promotion of the wide use of JCPs with patients who have experienced crises. It is important to continue to research JCPs through impact studies that include family members.

## Introduction

Relapse can lead to harmful consequences for people with severe mental illness and to a reduction in decision-making capacity (1). Therefore, it is important to prevent relapse to ensure patients' security and recovery. Patient-centered care as well as the recovery model have promoted patients' active participation in their care process. A recent systematic review and meta-analysis evaluated the effectiveness of self-management interventions for people with severe mental illness (2). The results showed a positive impact on total symptom severity, negative symptoms, and depression and anxiety symptoms. Although no significant impact was found on relapse and readmission, a reduction in the average length of hospital stay was reported (2). Among interventions fostering self-management, the Joint Crisis Plan (JCP) has received growing interest in clinical and research settings. Studied mainly over the past decade in English-speaking countries, the JCP is a type of psychiatric advance statement that describes how to recognize early signs of crisis and how to manage crises (3). The first pilot study on the impact of JCPs on rehospitalizations, especially without consent, and health costs showed encouraging results (4). Nevertheless, randomized controlled trials did not confirm these results (5–9). Studies that have evaluated the impacts of JCPs have reported many other advantages, such as positive perceptions of the tool by a majority of patients as well as the ability of the tool to reinforce patients' feelings of security and to allow them to gain better control of their health and life, develop better knowledge of their illness, have greater motivation to maintain their treatment, and create stronger therapeutic relationships (5, 6, 10). Concerning providers, a feasibility study showed that they perceived better medication compliance and better therapeutic relationships due to a readjustment of the balance of power between patients and providers (11). Thus, the use of the JCP is thus not disputed since its advantages go beyond avoiding coercion and hospitalization. Rather, the JCP implementation process and resistance to its use are questioned and investigated. These factors have been reported as potential biases in randomized controlled trials (5). This result highlights the necessity to consider the context in which the JCP is used specifically in terms of the training and the institutional measures needed to ensure its efficiency (12). The necessity for such a consideration has been recommended by studies that focused on the identification of obstacles to the use of advance statements (3, 13). In Switzerland, there is a growing interest in implementing interventions based on the recovery model. Successful experiences are sparse and strongly depend on the culture of the concerned organization. In the Canton of Vaud, a state in western Switzerland, JCP implementation began at different degrees in various care settings, such as hospitals, ambulatory and community settings, and sheltered accommodations (14, 15). In 2015, a local report issuing recommendations on the quality and coordination of care recommended introduction of the JCP, especially upon patient hospital discharge (16). This stage is recognized as a critical period in terms of risk of relapse, discontinuity of care, and suicide (17–19). To our knowledge, no study has been conducted on the content of JCPs in French-speaking countries. Moreover, the studies carried out on JCPs have mostly included outpatients. This study fills this gap in the scientific literature.

The purpose of the present study was to describe the content of JCPs and how they are perceived by patients, their relatives, and the professionals using a mixed method. This article focuses on the content of the psychoeducational items of the JCPs (crisis triggers, crisis manifestations, and coping strategies) and the perceived benefits and limitations of the tool according to the patients, their relatives, and the providers.

## Methods and Materials

### Design and Data Collection

The study used an exploratory mixed, sequential method. First, JCPs completed in hospital were retrospectively collected in 2016 and from January to April 2017 in other settings. Qualitative and quantitative content analyses were performed (PF and PL). Based on the relevant themes identified in the analysis as well as on the literature review, a semi-structured flexible topic guide with open-ended questions was developed by the research team (PF, PL, and CS). Two main topics were explored through the following questions: “In your opinion, what are the added values of the JCP?” and “According to your experience, what are the limitations of the JCP?” Two close-ended questions that measured the usefulness of and satisfaction with the JCP as perceived by patients, providers, and family members completed the interview. Specifically, to measure JCP usefulness, participants were asked, “To what extent is the JCP a useful tool for you, for providers, and for family?” Participants answered the same questions for themselves and for the other actors. Satisfaction with the JCP practices was measured for each participant with the following question: “To what extent are you satisfied with the writing, application, and revision processes of the JCP?” Both perceived usefulness and satisfaction were measured on a 10-point rating scale ranging from 0 (“not at all”) to 10 (“completely”). Finally, for each participant, the following data were gathered: gender, age, diagnoses, years of illness, and duration since completion of a JCP for the patients and gender, age, profession, years of practice, and training on the JCP for the healthcare providers. Interviews were audiotaped, transcribed (MM), and analyzed using content analysis method and descriptive statistics.

### Data Analysis

A hybrid approach was used according to Creswell (20). Both inductive and deductive content analyses of the whole qualitative material were conducted using NVivo 12 software. The deductive process was guided by the JCP pre-defined items (current care and treatment, crisis triggers, crises manifestations, strategies to deal with crisis, contact persons in case of crisis, preferred care and treatment in case of crisis, care and treatment to avoid) and by the different topics questioned in the interviews (how, by whom, and when were the JCPs written; how was the process structured; benefits and limits of the JCPs; and facilitators and obstacles of implementation). Then, an inductive analysis was performed, and themes arising from the data were coded until saturation was reached. The obtained themes were condensed and combined, and then described according to their frequency, saturation, and intensity. Coding strategies and the interpretation of data were cross-checked by independent researchers. Two researchers (PL and PF) independently coded a set of JCPs and interviews and compared their results. Any disagreement was discussed with a third experimented researcher and the peer practitioner (CBe) until agreement on the meaning and the application of each code was reached. Thereafter, the two independent researchers coded another set of interviews, and the interrater reliability was calculated (Cohen's kappa). This process was repeated three times, and a total of 40 JCPs and 10 semi-structured interviews were double-coded. An average Cohen's kappa of 0.97 for the JCPs and of 0.95 for the interviews was reached. Thus, the interrater reliability was excellent. The remaining JCPs and interviews were then coded by only one researcher.

Descriptive statistics were used to analyze the participants' profiles and characteristics. For the close-ended questions on perceived usefulness of and satisfaction with JCPs, mean scores were calculated. All statistical analyses were performed using IBM SPSS 26.

Finally, results were discussed with an integrative approach with the research team (PF, PL, CS, and MM) in a larger setting including community psychiatric experts and confirmed researchers with expertise either in quantitative (CB, JF, and PG) or in qualitative methodology (CBe and BS).

A peer practitioner (CS) was involved in the whole process, especially during data collection and findings analysis.

### Participants and Recruitment

Because JCPs have only recently been introduced in the local area and are thus far from a systematic implementation, a convenience sampling was used. Concretely, recruitment took place in various clinical settings that already used JCPs. At the time of the study beginning, JCPs were implemented in hospitals, community setting, as well as in sheltered accommodation. The concerned institutions were identified with the aid of the Public Health service, Department of Health and Social Action of the canton of Vaud. These were approached by the research team, and if managers were interested, they provided a written contract as a participating institution. Each institution was then asked to make a rough estimation of the intern prevalence of the JCP. Since the study was explorative, there was no prerequisite requirement in terms of the number of JCPs needed to be included in the study. The JCPs were retrospectively collected and sent to the researchers after anonymization.

In the hospital setting, clinical and sociodemographic profiles of the patients whose JCPs were included in this study were obtained from standardized statistical data routinely collected for the Federal Office of Social Insurances (OFS). In the other clinical settings (community and sheltered accommodations), these data were collected using the brief version of the Client Sociodemographic and Service Receipt Inventory (CCSRI).

Purposive sampling was used to select the patients and the providers for the interviews according to the principle of maximum variation in terms of sociodemographic characteristics, clinical profile, and environmental background. Thus, the main inclusion criterion was to have experienced the use of a JCP. Recruitment continued until saturation was reached. Interviews were conducted in a location chosen by the participants. For patients, this was mainly their place or an office at an ambulatory consultation. For professionals, this was mainly their workplace. Two of the authors (PL and PF) and the peer practitioner (CS) conducted the interviews. The interviews lasted on average 1 h.

Access to existing routine institutional records without explicit consent was previously granted by the Human Research Ethics Committee of the Canton Vaud (protocol #2016-00768). An addendum to the initial study protocol including information and consent forms and the interview guide was also approved. Managers of the participating institutions were contacted again and asked to provide a list of potential participants (patients, family members, or providers), who received an information sheet on the study. Those who gave oral consent after a time of reflection were introduced to the researchers through their clinicians. Willing participants then provided written informed consent.

## Results

### Sample

A total of 184 JCPs were obtained: 100 from hospitals, mainly completed upon hospital discharge; 29 from ambulatory settings; and 55 from community settings (nursing homes). Seven different models of JCPs were identified. Nevertheless, each JCP included several similar sections, which allowed content analysis to be carried out. JCPs often consist of an A4R two-sided sheet with questions that allow the investigation and description of the context in which a crisis may occur and the actions to undertake to manage it. Although different JCPs were used in the healthcare network, they all cover the following areas: the reasons for the current crisis; the triggers and manifestations of the crisis; the strategies to deal with the crisis; the persons that the patient can call to receive help; the care and treatments desired and to be avoided in the case of a crisis; and, finally, the social measures to undertake to preserve the interests of the patient. The patients who completed the JCPs were mainly female (52.5%), single (56.9%), unemployed (74.6%), and with a diagnosis of substance use disorders (45%), psychosis (36%), mood disorders (15%), anxiety and personality disorders (3%), and dementia (1%).

For the semi-structured interviews, 12 patients and 12 providers were recruited to reach data saturation. The mean age of the patients was 45.8 years, and the majority were females (9/12). All of them had suffered from mental illness for more than 10 years. Their diagnoses were mood disorders (4), psychosis (3), anxiety disorder (1), personality disorder (1), substance use disorder (1), Asperger disorder (1), and eating disorder (1). The majority had a PCC for <1 year. The number of interviewed patients per setting was as follows: 1 from a hospital, 5 from sheltered accommodations, and 6 from ambulatory settings.

The mean age of the providers was 36.9 years, and the majority were females (11/12). Six of them had between 6 and 10 years of professional experience. Eight nurses were part of the sample, and the majority (9/12) had received no training on JCPs.

The inclusion of family members in the study was not possible. First, it was difficult to identify family members, even though the main caregiver was often related to the patient. Only few family members had actively participated in the development of a JCP. Family members who were contacted refused to participate in this study because of current conflict with the patient, illness, or a lack of willingness to be involved in the JCP.

### Content of Existing JCPs

Regarding the content of existing JCPs, only data related to psychoeducational aspect items are presented in this article. The content analyses led to three main themes, *Crisis triggers, Crisis manifestations*, and *Strategies to deal with crises*, and 15 subthemes. These aspects particularly highlighted the patients' contribution in promoting their health. In the creation of JCPs, these aspects were initially completed by patients themselves, with providers taking a non-directive approach. Patients' points of view were solicited and prioritized. In addition, patients' points of view were the most referenced in JCP practices. For each category, the number of references and illustrations with verbatim quotations are provided.

#### Crisis Triggers, Crisis Manifestations, and Strategies to Deal With Crisis

The inductive content analyses led to the identification of the five subthemes for crisis triggers. Contents are presented in Table 1. Crisis triggers were present in 167 JCPs, with a total of 723 references (4.3 on average for each patient). More than 2/3 of JCPs had content classified in the “other” category. The very personal and precise content of these testifies to their singularity. The second largest number of crisis triggers were relational, followed by social and health-related ones.

**Table 1:** Contents analyses of joint crisis plans.

**Crisis triggers**	**JCP (*N* = 184)**		**Verbatim quotations**
	***N***	**%**	
Relational	101	55%	“Conflicts with my family and at work”
Social difficulties	78	42%	“Anything to do with work, unemployment and insurance”
Health concerns	56	30%	“Auditory hallucinations with commands to hurt myself”
Other	104	57%	“Scenes of street violence”
No information	17	9%	
**Crisis manifestations**	**JCP (*N* = 184)**		**Verbatim quotations**
Symptomatic	157	86%	“Elements of persecution (surveillance, microphones, cameras)”
Behavioral	136	74%	“I don't answer the phone anymore; I barely listen to messages”
Relational	38	21%	“I need a constant presence”
Other	109	59%	“Emotions on edge”
**Strategies to deal with crisis**	**JCP (*N* = 184)**		**Verbatims quotations**
Engaging in activities	158	86%	“Listening to music”
Interacting with others	108	59%	“Call a friend or go drink a coffee”
Taking medication	72	39%	“Temporarily increase the Abilify”
Managing emotions	36	20%	“Do a mindfulness exercise for 5 min”
Using drugs	11	6%	“Drink alcohol alone”
No information	6	3%	

##### Relational Triggers

Relational triggers were reported in 55% of JCPs (*N* = 101). In more than half of the cases, they were related to conflicts with family members, e.g., “family conflicts;” with partners, e.g., “relational conflicts with my ex-husband;” or in work relationships, e.g., “conflicts with my colleagues.” Illness or death of a close person was reported 18 times, e.g., “death of a person I love,” and the next most common was a feeling of loneliness (*N* = 14) and sentimental break-ups (*N* = 7). An overstimulating environment was also mentioned (*N* = 6).

##### Social Difficulties

Social difficulties were reported in 42% of JCPs (*N* = 78). The majority were related to unemployment at work (*N* = 52), such as “have not found traineeship nor work” and “uncertainty at work.” A difficult work environment and too many responsibilities were also expressed: “too many responsibilities” and “too many tasks in a very short time.” Financial preoccupations (*N* = 37) were also listed, e.g., “My legal guardian does not pay my bills.” Housing instability (*N* = 13), insecurity about social status (*N* = 7), and justice problems were the least common social difficulties (*N* = 6).

##### Health Concerns

Health concerns were the least reported triggers and were present in 30% of JCPs (*N* = 56). Psychiatric symptoms, such as “anxiety,” “flashbacks,” “suicidal thoughts,” and “sleeping troubles,” were the most frequently expressed (*N* = 46), followed by somatic symptoms (*N* = 17), including “headache” and “more important dyspnea.” Difficulties with medication (*N* = 10), general anxiety (*N* = 8), emotional disorders (*N* = 2), and hospital discharge (*N* = 2) were also reported.

##### Other Crisis Triggers

Other crisis triggers covered a variety of topics and were present in 57% of JCPs (*N* = 104), e.g., example, “people's noises in the street,” “being in society when people are drunk,” “public transports,” and “fear of failure.”

About crisis manifestations, the inductive content analyses led to the identification of four subthemes. Contents are presented in Table 1. Crisis manifestations were present in almost all JCPs (*N* = 182), with a total of 1,588 references (8.7 on average references for each patient). The largest number of manifestations were symptomatic, followed by behavioral. More than 1/2 of JCPs were classified in the “other” category. Finally, 21% JCPs reported relational manifestations.

##### Symptomatic Manifestations

Psychiatric symptoms were reported in 86% of JCPs (*N* = 157) and included the main psychiatric disorders. Depressive symptoms (*N* = 228) followed by somatic symptoms related to anxious disorders (*N* = 228) were the most depicted followed by manic symptoms (*N* = 64), for example, “loss of pleasure,” “difficulty breathing,” and “racing thoughts.” Suicidal ideations (*N* = 63) and psychotic symptoms (*N* = 59), such as “a very strong and uncontrollable desire to leave life” and “visual hallucinations (animals in the sky),” were also observed. Finally, self-harm was reported six times.

##### Behavioral Manifestations

Behavioral manifestations were reported in 74% of JCPs (*N* = 136). Withdrawal behaviors were the most common (*N* = 105) and were present at varying degrees up to avoidance of the outside world, for example, “sit back in my bedroom, I don't answer the telephone” and “I do not go outside anymore.” Other behaviors, such as aggressive behaviors (*N* = 88), were depicted as “clastic crisis.” The cessation of domestic and professional activities was reported 29 times: “I don't go to work and just stay home” and “I don't take care of myself and my belongings.” Drug use (*N* = 23) and disordered eating habits (*N* = 14), for example, “I eat compulsively all day” and “endangering with drug use,” were also noted.

##### Relational Manifestations

Relational manifestations were reported in 21% of JCPs (*N* = 38) and included behaviors aimed at interacting with others, such as “I ask a lot of help to my colleagues.”

##### Other Manifestations

Other manifestations were present in 59% of JCPs (*N* = 109) and referred to personal feelings and emotions, such as “I'm more sensitive to what surrounds me” and “I feel dirty, great malaise.”

Finally, the inductive content analyses for strategies to deal with crisis led to the identification of the six subthemes. Contents are presented in Table 1. Strategies to deal with crisis were present in almost all JCPs (*N* = 178), with a total of 970 references (5.4 references on average for each patient). Engaging in activities and interacting with others were the most frequent, followed by taking medication and managing emotions.

##### Engaging in Activities

Engaging in activities was reported in 86% of JCPs (*N* = 158). The following activities were represented: walking, listening to music, working out, watching a movie or reading a book, taking a shower or a bath, and performing creative activities. Example quotations were “walk outside” and “I watch funny things on television.” Withdrawal was considered an activity insofar as it was intentional and was reported in 24 JCPs: “I sleep to forget” and “I isolate myself in my room because when I isolate myself, I get better.”

##### Interacting

Interacting with another person was present in 59% of JCPs (*N* = 108), and a minimum of two persons were mentioned. The most frequently mentioned persons of contact were family members (*N* = 79), followed by professionals (*N* = 71), for example, “I can call my father” and “I call my nurse or my psychiatrist.” Sometimes (*N* = 37), no specific person was identified, such as “increase social interactions.”

##### Taking Medication

Taking medication was reported in 39% of JCPs (*N* = 72) and included, for instance, anxiolytic drugs (*N* = 37) followed by neuroleptics (*N* = 10): “take a reserve of Anxiolit” and “take neuroleptics again.” The use of medications (*N* = 21), such as somatic medications or specific substances like melatonin, was also described in terms of adaptation of medication, such as “reevaluation of the medication.” Performing specific emotion management techniques (*N* = 36) and taking psychoactive substances (*N* = 11) were the least frequently mentioned strategies.

### Interviews With Patients and Providers

Regarding the content of the interviews, only data related to benefits and limits are presented in this article. The content analyses of the benefits led to seven main themes: JCP as a supportive and reassuring tool; JCP as a valuable tool for continuity and coordination of care; JCP as a tool to reduce the sense of urgency; JCP as a tool to prevent hospitalizations; JCP as a psychoeducational tool; JCP as a tool to empower therapeutic relationships; and JCP as an advocating tool of patients' preferences and choices.

The content analyses of the limits led to eight main themes: lack of perceived usefulness; patients' refusal to talk about crisis again; lack of information and training; difficulty to adopt an asymmetric caregiver–patient relationship; anosognosia and acute symptomatology; lack of accessibility; absence of guidelines; and intellectual disabilities, intoxication, and cognitive impairments.

#### Perceived Usefulness and Satisfaction of the JCP

**Figure 1:**
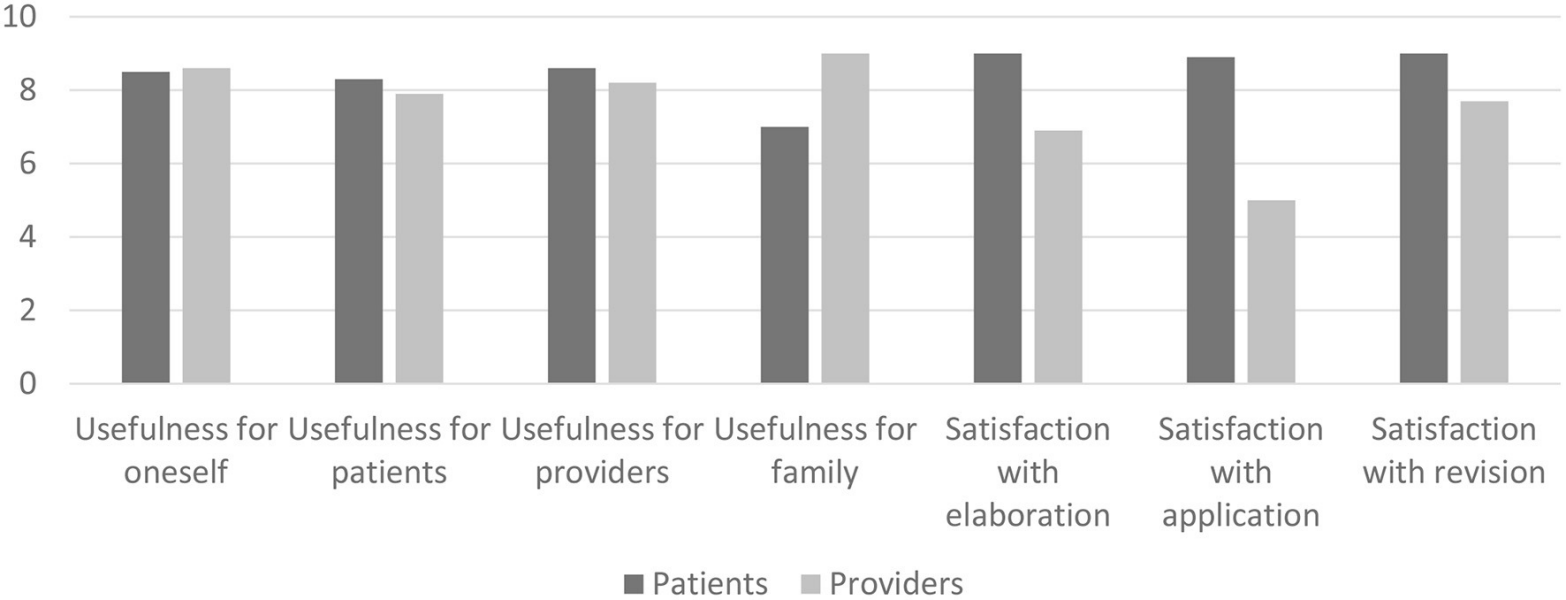
Perceived usefulness of and satisfaction with the JCPs.

Rating scales showed that the JCPs were perceived as useful for patients and providers. Figure 1 shows the perceived usefulness of and satisfaction with JCP practices (*N* = 12 patients and *N* = 12 providers). Satisfaction with the elaboration, application, and revision of JCPs was evaluated because these three steps were identified as an integral part of the JCP practice process by all participants, as revealed by the content analysis of the semi-structured interviews. Scores were reported on a 10-point Likert scale ranging from not at all (0) to entirely (10). The mean scores are presented.

The JCP was perceived as a very useful tool for patients and providers, with scores ranging from 7.9 to 8.6. The dimensions with the highest scores among patients were “usefulness for patients” (8.3) and “usefulness for providers” (8.6). Providers thought the tool was more useful for family than for patients, with respective scores of 9 and 7.

Concerning the JCP practices, patients were very satisfied, with scores ranging from 8.9 to 9. The highest score was observed for the dimensions “satisfaction with elaboration” and “satisfaction with application” with respective scores of 9 and 8.9, while providers rated these two dimensions with scores of 6.9 and 5, respectively.

#### Perceived Benefits and Limitations of the JCP

##### Benefits

Six main benefits were perceived by both patients and providers (*N* = patients and *N* = 12 professionals). The results were derived from analysis of the semi-structured interviews. For each benefit, frequencies and illustrations with verbatim quotations are provided. The most common benefit reported by participants and especially by professionals was that JCP is a supportive and reassuring tool when crisis occurs (*N* patients = 8, *N* professionals = 10).

“*I think it's interesting to put it in black and white, to see where our resources and our weak points are and which situations could stress us or not, to analyze these things a bit oneself. I think it's really concrete. For me that's what's important; it*'*s very positive.”*

*Professional*: *The fact that the patient can have concrete tools and strategies to deal with symptoms that may occur in case of crisis and know concretely who to contact and what to do, that*'*s an important benefit*.

Although no family member directly took part in the interviews, patients and professionals reported that families perceived these same benefits.

*Patient*: *It is precisely the fact that now the people who are around me, whether family or therapeutic entourage, can act of their own free will in case of problem*.

*Professional*: *Families*' *feedback is very positive too. It often is a relief when they learn that a JCP has been made*.

The JCP helps ensure the transmission of information and makes crisis management easier because it provides practical information, for example, about the persons to contact in case of a crisis, and thus contributes to an effective coordination of intervention (*N* patients = 5, *N* professionals = 7). As the JCP is created with patients, it favors patients' empowerment.

*Patient: Having something like written support really allows you to focus on the problems you are going through. There is no way to cheat by saying, “Maybe it's not that; it's because you're stressed”… So, we know it comes from the illness. And it allows us to make decisions*.

*Professional: It's something nice because we have phone numbers to contact people quickly. We don't need to look numbers up in telephone directories or whatever. It's accurate; we have very clear information, and the patient being the actor of this document is important*.

In case of crisis, the JCP reduces the sense of urgency, especially for patients for whom the intensity and variability of symptoms are important and disturbing (*N* patients = 6, *N* professionals = 6). Furthermore, it prevents hospitalizations (*N* patients = 1, *N* professionals = 1).

*Patient: It's really a very good tool because precisely with psychological problems like depression, we are overwhelmed by parasitic and sometimes unreal thoughts, so it's good to have something that puts us back on the ground*.

*Professional: Especially for the most complex or unstable situations in which patients can move through different states in the same day or in the same week. So it can help to have an approach… Well, less of a sense of urgency that can be disturbing*.

*Patient: It prevents me from being hospitalized*.

The JCP was perceived as a psychoeducational tool that helps patients and professionals talk about illness and symptoms (*N* patients = 6, *N* professionals = 6). The JCP makes communication about illness easier and helps patients and professionals share their expertise about how to manage it.

*Patient: It helps to identify problems… like behavioral disorders that are normal for me, but when I identified them, I realize that it's because I'm sick*.

*Professional: We can really work on how they (patients) perceive their illness and their behaviors and the strategies to deal with them. So, it's really a good gateway to delve into these aspects. Of course, they (strategies) are mentioned in interviews but not always in detail. The JCP helps to do that*.

In addition to facilitating communication about illness, the JCP empowers therapeutic relationships and partnerships because it allows the consideration of patients' preferences and choices about treatments to manage illness (*N* patients = 5, *N* providers = 1).

*Professional: It is a good tool that really fosters the therapeutic relationship and can sometimes enhance complex topics such as medication compliance. For example, the patient may not be able to see the usefulness of the treatment, and we can discuss the topic with him/her through the joint crisis plan*.

*Patient: So, I think it's something very useful because we as patients express ourselves, it's not the doctor who tells us to take this or that drug*.

*Professional: It is the patient who is the actor of this document, and that is important. It's something I'm sensitive to. It is really their will that's important to fully respect*.

*Patient: I know that if I am hospitalized, I will receive the treatment that I would have discussed with my contact in the Joint Crisis Plan and that would somehow reassure me*.

##### Main Perceived Limitations

The following section describes the limitations of the JCP perceived by the interviewed patients and professionals (*N* = 12 patients and *N* = 12 professionals). The implementation of a PCC must remain a voluntary process. Both patients (*N* = 3) and professionals (*N* = 8) reported the lack of perceived usefulness as an important limitation of JCP as was the patients' refusal to talk about a crisis, which could lead to the emergence of painful emotions related to traumatic events (*N* professionals = 4). The lack of information and training on the JCP and the adoption of a posture that contributed to an asymmetric caregiver–patient relationship were also highlighted by two professionals.

*Patient:…You know, it's just stuff I can't answer, because since I was a kid, I've been placed, I've been beaten, I've never asked for help when I had care problems, so there are lots of… I don't ask, you understand it's difficult*…

*Professional:… I typically think of caregivers' resistance, so in so far as it is a relationship that is more balanced, more symmetrical, and it can be problematic; it can be scary to think that ultimately, it is the patient who decides what to do with him/her. I also find that it is a step in our profession to work on collaborative aspects. I can't speak for them (patients), but I think the fear is of losing power, and being with an all-powerful patient, it's like a phobia, a fear of caregivers*.

Anosognosia of the disease and acute symptomatology were also considered as limitations to the elaboration and application of the JCP. During its application, the intensity of symptoms may compromise crisis management because it may reduce introspection and action capacities. Moreover, the crisis is a critical and painful moment that is difficult to manage for patients.

*Patient:…Because I wasn't very well yet, so it was difficult for me to get the perspective and the necessary perspective to know what… when you're not well, you're in, you can't really determine*…

*Professional:… It depends on the patient's level of awareness of the disease. It makes things more difficult or easier, and all the work we've been able to do together on psychoeducation around the disease and symptoms depends on where we are in our common work*.

The lack of accessibility of the JCP is another limitation, particularly during its implementation. No official guidelines exist concerning the electronic storage of JCP, making its transmission within the care network extremely complex, especially toward emergency services.

*Professional: It may not be used because…information, accessibility, people, well that's how you know the person has made a JCP, where it is, how you get to know it; depending on the context too, it's not simple*.

Finally, some mental disorders, such as intellectual disabilities, cognitive problems, or substance abuse, were also perceived as jeopardizing the use of the JCP (*N* patients = 3, *N* professionals = 2).

## Discussion

### Contents of JCPs Related to Crisis

The purpose of the current study was to employ a mixed method approach to describe the content of existing JCPs and their perceptions by patients and professionals. Documented in numerous and varied ways, the contents of JCPs are pertinent and relevant. Our findings indicate that patients have good knowledge of themselves and their illness. Regardless of the problem, patients and professionals identified triggers, manifestations, and strategies to manage crisis. Each section of the JCPs provided essential and personal information for patients with two main goals: manage the crisis and stay healthy. This latter was a priority for patients, while professionals prioritized crisis avoidance and management.

Content related to crisis triggers primarily included relational and social difficulties, followed by health concerns. This finding highlights the importance of considering these difficulties in the therapeutic work, which should not focus only on medical concerns as patients still often report (21). More than half of the triggers are patient-specific and cannot be categorized, indicating that a disease- and symptom-centered reading of a crisis is not sufficient and does not reflect the patient's concerns. It is therefore important to establish a personalized crisis plan that allows these concerns to be considered. Otherwise, the plan would be perceived as non-specific and not reflective of the patient's experience.

Regarding content related to crisis manifestations, symptoms linked to depression were broadly depicted, followed by somatic symptoms. This finding highlights that psychiatric disorders have a close relationship with the incidence of somatic issues, and vice versa. Withdrawal must first be seen as a coping strategy, but it must lead patients and professionals to determine what to do if withdrawal lasts too long and become a symptom. It is necessary to discuss when a proactive attitude should be adopted to ensure that care continues to be delivered. More than half of the manifestations could not be categorized and were, similar to triggers, patient specific.

In JCPs, the most frequently mentioned strategies to deal with crisis were social-related: engaging in activities or interacting with others. This finding highlights the strong impact of social conditions on mental health. This is consistent with the concept of recovery, an essential part of which is playing a social role and being connected with others. Medication or emotion management strategies were reported in less than half of JCPs. This finding raises questions about the perceived usefulness of the usual clinical strategies adopted in crisis management. If these clinical strategies are to be used more effectively, it is necessary for patients to become more knowledgeable about their disorders and their treatment, and to have more autonomy in this area, particularly through psychoeducation. Finally, for a minority of patients, the use of drugs or alcohol constitutes a crisis management strategy. This point probably requires a discussion to examine how effective this strategy is and what the alternatives are.

The content related to triggers, manifestations, and strategies to manage crisis incorporates broader biopsychosocial aspects than are usually considered in care. These categories were well-documented in the existing literature, except for crisis triggers, which are not systematically reported in JCPs despite their essential role in preventing crises (22, 23). JCPs integrate essential elements of recovery, such as hope, social relationships, empowerment, and prioritization of personal resources. Patients seem more comfortable with this vision of recovery than professionals.

### Patient and Professional Perceptions on JCPs

The JCP was perceived as a very useful tool by patients and professionals, with both groups giving high and relatively similar scores, except for perceived usefulness for families, which was lower for patients. Although it was planned to include family members in the sample, this was not possible. Indeed, family members were not mentioned in the majority of the JCPs, and the two individuals who were contacted declined to participate. These results question the role of family in crisis management and the reasons why family members are not involved in JCPs. Some patients also noted that their JCPs were too personal to be shared with relatives. Our inability to question anyone was a result in itself, indicating the actual state of the art. Concerning the JCP practices, patients were more satisfied than professionals with its application. This result highlights that patients use the JCP as a supportive and reassuring tool in daily life and not only in case of crisis, whereas professionals are more concerned with the JCP when crises occur.

Many benefits reported by both patients and professionals highlight that interest in the JCP goes beyond a desire to avoid hospitalizations, as also found in previous studies (5–10). The following benefits were reported: the improvement of the therapeutic relationship, the honoring of patients' choices and wishes by professionals and a greater sense of control of illness (5, 7, 24). Respect for choices and preferences was more frequently perceived by patients. This finding indicates the need to raise awareness among professionals of the importance of respecting patients' choices and preferences and of methods to achieve this goal, such as shared medical decision making. Previous studies have mainly included patients suffering from psychosis, bipolar, or borderline personality disorders, whereas the current study included patients with a broader range of diagnoses. It can be hypothesized that these benefits are not linked to diagnosis and apply to a large range of patients in the event of a crisis. Furthermore, the JCP analyses were retrospectively conducted, and they highlight that the JCP can be used for all types of patients in varied care settings, including hospitals. For hospitalized patients who have been admitted involuntarily, the JCP may contribute to decreasing pressure on hospital beds and improving the use of socio-sanitary resources.

Findings about JPCs' limitations are sparse in the existing literature. As in previous studies, the present results indicate that there is a lack of training on JCPs for professionals and difficulty of shifting from a paternalistic to a more balanced professional–patient relationship, which constitutes an important challenge for providers because it is rooted in culture (5, 11). In fact, the development of a more balanced relationship calls for professionals to share or cede significant power to the patient. Indeed, the JCP requires professionals to change their attitudes toward partnership with patients; to become open to events, triggers, and strategies outside the usual clinical framework; and, finally, to increase their ability to overcome the obstacles to the development of a JCP in the therapeutic relationship. The lack of accessibility of JCPs was also reported (24). This can be problematic in the event of a crisis when the intervention of professionals is necessary and may explain why professionals had a lower level of satisfaction than patients with the application of JCPs. This is particularly true when the intensity of symptoms may limit patients' capacity for introspection and action. In these cases, it is important that professionals have knowledge of patients' wishes for treatment.

## Strengths and Limitations

The formation of a multidisciplinary research team made it possible to bring together different clinical and research expertise for this study. In addition, the presence of a peer practitioner ensured that the patient's perspective was represented. The team met at each major stage of the research, including discussion and exchange of opinions on data collection and outcomes. This approach increased the internal validity. In addition, the content analysis of the JCPs and the interview transcripts were double coded to ensure scientific rigor, with statistical tests demonstrating excellent inter-rater reliability. Finally, to our knowledge, this is the second French-language study on this topic, the first one at an international level conducted with real-world data, and including hospitalized patients, who were therefore potentially sicker and more unstable than the participants of previous studies. The number of JCPs included in the study was larger than that in all other studies, thus optimizing the external validity.

Regarding the limitations, the study did not consider all practices in the field. Only patients and professionals who used a common crisis plan were interviewed, which constitutes a possible selection bias. It would also be necessary to have data on who refused or declined to develop a JCP. In the context of the study, the JCPs were not systematic. All diagnoses were represented, so there were other elements that could have been an impediment to their development. Furthermore, as the participants volunteered for the interviews, it is possible that only those interested in the subject and who perceived benefits participated. In addition, a potential memory bias may have led participants to overlook the difficulties in developing the plan, especially if the JCP was currently perceived as useful. Despite the important number of participants to the interviews for a qualitative study, the transferability of the results may be limited by the local context.

## Conclusion

The JCP is perceived as a very useful tool by patients and professionals. For patients, the JCP sometimes seems to be used as a tool for staying healthy, whereas professionals seem to more directly link the JCP to advance directives. The links between these two tools need to be clarified. It seems important to raise the awareness of healthcare professionals about patients' interest in JCPs and the importance of discussing the extent to which patients can play an active role in health promotion before a crisis occurs. In all cases, it is important to identify the individual specificities of the disorder. Professionals must take these specificities into account without focusing solely on crisis management strategies with medical connotations. The elaboration of a common crisis plan must be integrated into overall psychotherapeutic work. It is a time-consuming process that requires patients to overcome avoidance of the illness to promote their awareness and gain power to act on it. The elaboration also involves negotiating medical and non-medical interventions with consideration of the patient's choices and preferences. A key question is how to systematize or prioritize the development of a JCP. With the current state of knowledge, there is a paradox between the perceived relevance of the JCP in a wide field of use and its low frequency of use. The present study was conducted in a naturalistic context, and the results highlight that the JCP may be used with patients suffering from a large variety of psychiatric disorders in different care settings. The JCP had only recently begun to be implemented at the time of the study, and it is important to continue to perform research with impact studies on a larger range of variables, including recovery in naturalistic settings; to include family members in studies; and to measure and document implementation processes. The promising results of this study encourage the wide promotion of the use of JCPs for patients who have experienced crises. In practice, the issue of universal access to JCPs needs to be resolved. On the one hand, broad access is needed for the use of JCPs in crisis situations; on the other hand, obstacles to ubiquitous accessibility and confidentiality need to be overcome. The necessity for the JCP to be linked with the electronic medical record of the patient and to be directly accessible upon written consent of the patient appears obvious. The issue of the training and positioning of professionals as well as the integration of the JCP into therapeutic approaches must also be considered.

## Data Availability Statement

The raw data supporting the conclusions of this article will be made available by the authors, without undue reservation.

## Ethics Statement

The study has been reviewed and approved by the Commission cantonale d'éthique de la recherche sur l'être humain (Approval No. 2016-00768). The patients/participants provided their written informed consent to participate in this study.

## Author Contributions

PF conceptualized the study. PL and PF completed the same measures, analyzed and interpreted the data, drafted the first version of the manuscript, and conducted the thematic content analysis of the JCPs and interviews. PF, PL, and CS acquired the data and facilitated the recruitment of participants. MM transcribed the interviews and the JCP content. CBe ensured triangulation as an experimental researcher. JF and CBo made substantial corrections to the first draft of the manuscript. All authors made substantial contributions to the analysis and interpretation of data and critically revised the article for important intellectual content, approved the final version for publication, and agree to be accountable for all aspects of the work by ensuring that any questions related to its accuracy are answered.

## Conflict of Interest

The authors declare that the research was conducted in the absence of any commercial or financial relationships that could be construed as a potential conflict of interest.
